# *Cystoisospora belli*: A Cause of Chronic Diarrhea in Immunocompromised Patients

**DOI:** 10.4269/ajtmh.23-0012

**Published:** 2023-04-10

**Authors:** Rimjhim Kanaujia, Abhishek Mewara

**Affiliations:** Department of Medical Parasitology, Postgraduate Institute of Medical Education and Research, Chandigarh, India

A 32-year-old man presented to the outpatient department with intermittent diarrhea for the past 1 year. He had received several courses of antibiotics from various hospitals but continued to have recurrent episodes of loose stools. He was diagnosed with HIV infection 5 months earlier and was currently on anti-retroviral therapy. On physical examination, he was dehydrated and emaciated and had pallor with temporal and supraclavicular hollowing. His sodium and potassium were 136.4 mmol/L (reference range: 135–145) and 2.75 mmol/L (reference range: 3.4–4.8), respectively. He was administered intravenous fluids, and hypokalemia was aggressively replenished. His CD4 cell count was 270 cells/mm^3^.

**Figure 1.**
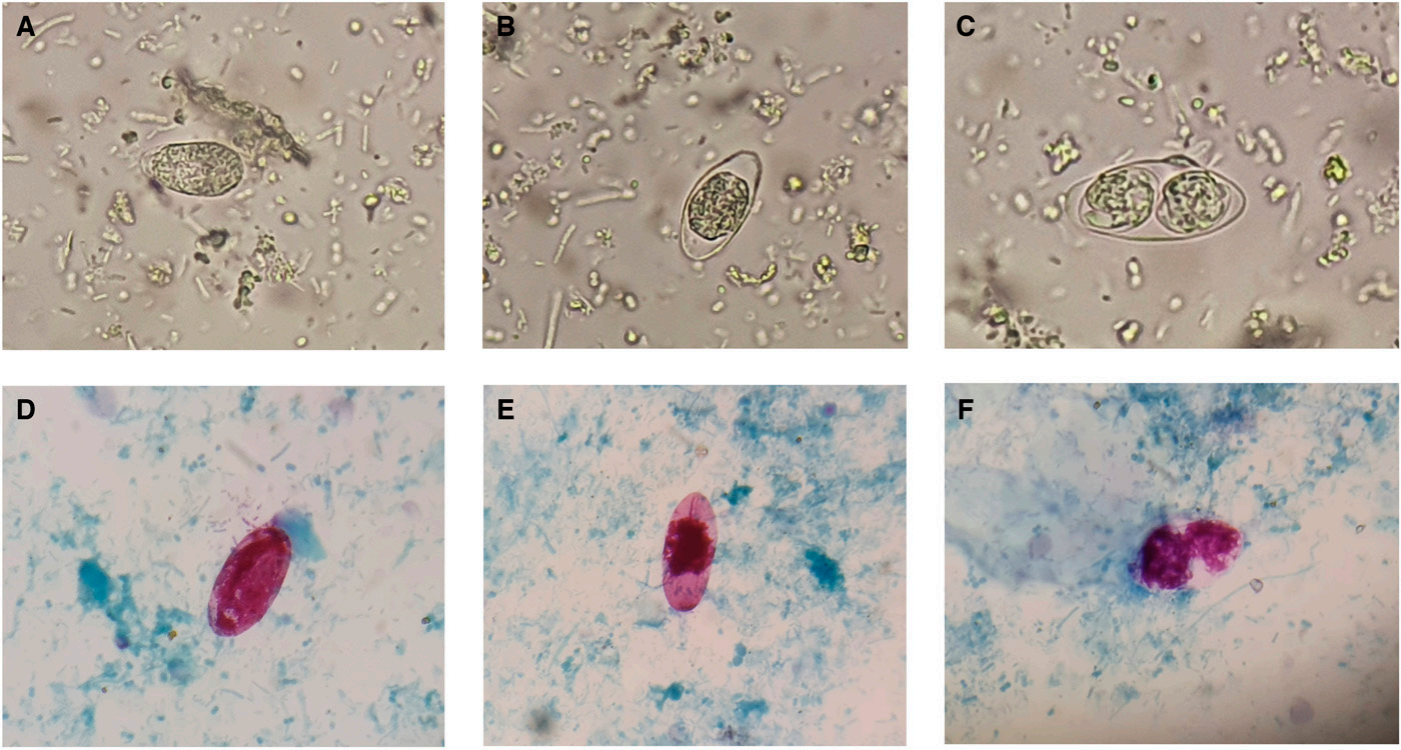
*Cystoisospora belli* in stool microscopy. Wet mount showing (**A**) immature unsporulated oocyst, (**B**) immature sporulated oocyst with single sporoblast, (**C**) mature sporulated oocyst with two sporocysts, and corresponding images in modified acid-fast stain (**D–F**).

The stool sample of the patient was sent for microscopic examination, which revealed *Cystoisospora belli* oocysts. The immature (unsporulated) and mature (sporulated) forms of the oocysts were seen in the wet mount (Figure 1A–C) and by modified acid-fast staining (Figure 1D–E) microscopy. He was started on trimethoprim-sulfamethoxazole (TMP-SMX) (160/800 mg) four times daily. The patient responded to the treatment and the diarrhea was resolved.

*Cystoisospora belli* infection occurs by ingestion of water or food contaminated with oocysts. It causes chronic diarrhea in immunocompromised patients.^1^ The stool examination may show the various forms of the oocysts—immature unsporulated (Figure 1A, B, D, and E) or mature sporulated oocysts (Figure 1C and F). Cystoisosporiasis is treated with TMP-SMX. However, relapse is common in patients with HIV who may require long-term maintenance therapy with TMP-SMX.^2^ Thus, follow-up of such patients is essential.
